# A Hybrid Ensemble Approach for Early‐Stage Diabetes Detection

**DOI:** 10.1049/htl2.70060

**Published:** 2026-02-14

**Authors:** Rachana Katuwal KC, Su Yang

**Affiliations:** ^1^ Department of Computer Science Swansea University Wales UK

**Keywords:** decision trees, diseases, health care, man‐machine systems

## Abstract

Diabetes has become a critical global health concern, particularly in regions where access to diagnostic facilities is limited. In this work, we propose a hybrid framework that combines extreme gradient boosting (XGBoost) and deep neural networks (DNNs) for early‐stage diabetes detection, using soft voting to generate the final ensemble predictions. The proposed framework was evaluated on two datasets: the widely used Diabetes UCI dataset and a newly collected dataset from Nepal. The ensemble method achieved 99% accuracy (ACC) with an area under the curve (AUC) of 1.00 on the Diabetes UCI dataset, and 91% ACC with a 0.96 AUC on the Nepal diabetes dataset, demonstrating its strong generalisability across distinct populations. Compared to individual models, the hybrid approach offered increased stability and a lower rate of false negatives, which is particularly important in clinical contexts. These findings highlight the potential of hybrid machine learning–deep learning models as cost‐effective, scalable and generalisable decision‐support tools for diabetes risk assessment.

## Graphical abstract

The study presents a hybrid framework combining XGBoost and deep neural networks with soft voting to enhance early‐stage diabetes detection. This approach improves accuracy, reduces false negatives and demonstrates strong generalisability across different populations, making it a promising tool for scalable clinical decision support.

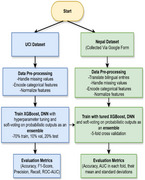

## 1 Introduction

Diabetes is a chronic condition that occurs when the body either fails to produce enough insulin or cannot use it effectively, leading to high blood glucose levels (hyperglycemia) [1]. If not properly managed, it can progressively damage major organ systems, particularly the heart, kidneys, eyes, nerves and blood vessels, increasing the risk of severe complications and premature mortality [2]. According to the International Diabetes Federation, 589 million adults are living with diabetes worldwide, with the majority (81%) residing in low‐ and middle‐ income countries. Alarmingly, an estimated 43% of adults with diabetes remain undiagnosed, and nearly 90% of these undiagnosed cases are in low‐ and middle‐income countries [3]. This surge is occurring particularly rapidly in these regions, where limited access to healthcare, high costs of diagnostic tests and low public awareness remain significant barriers to timely detection and effective management [4].

Early prediction of diabetes plays a vital role in mitigating long‐term risks and reducing healthcare costs. Traditional clinical tests, such as fasting blood glucose and HbA1c, while accurate, are often inaccessible or unaffordable in resource‐constrained regions. Leveraging non‐clinical, symptom‐based datasets provide a low‐cost and scalable alternative.

For example, the widely used Diabetes UCI dataset captures self‐reported features such as excessive thirst, frequent urination, sudden weight loss, fatigue, delayed wound healing and vision problems [5], enabling predictive modelling without laboratory measurements. Such approaches are particularly valuable in LMICs, where scalable and affordable diagnostic support tools are urgently needed. Over the past decade, conventional machine learning (ML) models such as Logistic Regression (LR), Support Vector Machines (SVM), K‐Nearest Neighbor, Naïve Bayes and Decision Trees (DT) have been applied to the UCI Early‐Stage Diabetes dataset for diabetes prediction, typically achieving accuracies in the range of 88%–96% [6, 7, 8]. To further improve predictive performance, researchers increasingly turned to ensemble methods such as Bagging, AdaBoost and Random Forest, which combine multiple weak learners. For example, Laila et al. (2022) reported that random forest achieved 97% accuracy on the UCI dataset, outperforming AdaBoost and Bagging in precision, recall and F1‐score [9]. Building on this, the field has recently advanced toward hybrid ensembles that integrate diverse algorithms to capture complex, nonlinear relationships in healthcare data. For instance, Bülbül (2024) proposed a Genetic Algorithm (GA) – Stacked Autoencoder (SAE) – Softmax hybrid, achieving 98.72% accuracy on the UCI dataset, surpassing traditional classifiers such as k‐NN, SVM and CNN [10]. Additionally, Ergün and İlhan (2021) reported 99.04% accuracy and 100% precision using a CNN model, demonstrating that near‐perfect results are attainable on this binary, symptom‐based dataset [11].

Despite these promising advances, most prior studies have evaluated their models exclusively on the UCI dataset, leaving their generalisability to new populations untested. This limits practical deployment, particularly in low‐resource settings where symptom prevalence and reporting behaviours may differ substantially.

In this context, we propose a hybrid ensemble of XGBoost and DNNs, designed to combine the structured learning capability of gradient boosting with the deep representation power of neural networks. Our contributions are threefold:

- Averaged fusion strategy: Integrates XGBoost and DNNs to capture both structured and nonlinear patterns
- Competitive UCI performance: Achieves 99.04% accuracy, matching the best‐reported results while attaining 100% recall, ensuring that no diabetic cases are missed in screening scenarios.
- Generalisability validation: Demonstrates 91% average accuracy on an independent Nepal diabetes dataset, underlining robustness and practical applicability in diverse populations.

## 2 Proposed System

The public UCI dataset from Kaggle, consisting of self‐reported early‐stage diabetes features [5], was first used for model development. Missing values were handled, categorical features were encoded into numeric form, class distributions were balanced and all features were normalised where required. To ensure strict separation between training and evaluation data, the dataset was split into 70% training, 10% validation and 20% testing before any preprocessing or model fitting was performed. Standardisation parameters for the DNN were learned exclusively from the training subset which was then applied to the validation and test sets, and the test set remained untouched until the final evaluation stage. These safeguards were implemented to eliminate the possibility of data leakage and ensure a fair assessment of model performance.

Model performance was evaluated using accuracy, ROC‐AUC, precision, recall, F1‐score and confusion matrices. Training and validation curves for both XGBoost (XGB) and the deep neural network (DNN) were monitored to verify stable learning and to detect potential overfitting.

Following the UCI evaluation, the same models—with identical hyperparameters—were applied to the Nepal diabetes dataset to assess external generalisation. This dataset required additional preprocessing due to bilingual responses in both English and Nepali. All Nepali entries were translated, categorical responses were encoded, and features were normalised using the same preprocessing pipeline. The Nepal dataset was evaluated using stratified 5‐fold cross‐validation to ensure robustness across heterogeneous subgroups, with accuracy and AUC recorded for each fold and aggregated as mean ± standard deviation. Soft‐voting was used to combine XGBoost and DNN predictions in both datasets.

**FIGURE 1:**
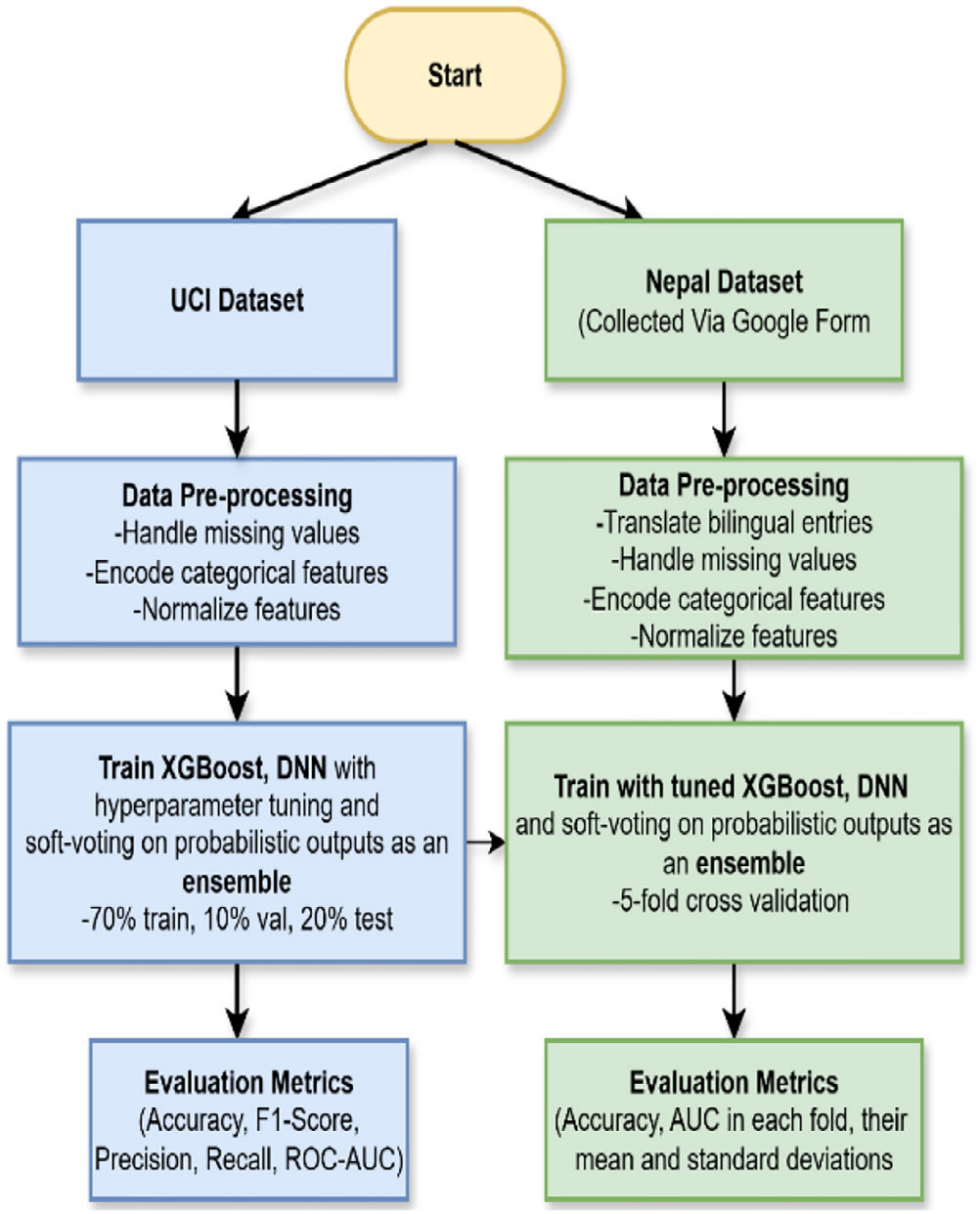
Illustrates the sequential stages of this research work.

The successive stages of this research are illustrated in Figure 1.

### 2.1 Datasets

This study utilises two datasets: the UCI Early‐Stage Diabetes dataset and a private dataset collected from Nepal.

#### 2.1.1 Diabetes UCI Dataset

The Diabetes UCI dataset is publicly available in the UCI Machine Learning Repository [5]. It contains 520 patient records with 17 attributes, including demographic information and self‐reported symptoms comprise of 61.5% non diabatic and 38.5% diabetic samples. An outline of the UCI dataset features is given in Table 1.

**TABLE 1:** Summaries the features of the UCI dataset.

Attribute	Data type	Possible values
Age	Numeric	16–90
Gender	Categorical	Female/Male
Polyuria	Categorical	No/Yes
Polydipsia	Categorical	No/Yes
Polyphagia	Categorical	No/Yes
Weight _loss	Categorical	No/Yes
Blurred vision	Categorical	No/Yes
Fatigue	Categorical	No/Yes
Alopecia	Categorical	No/Yes
Delayed healing	Categorical	No/Yes
Obesity	Categorical	No/Yes
Tingling numbness	Categorical	No/Yes
Family History	Categorical	No/Yes
Genital thrush	Categorical	No/Yes
Diabetic	Categorical	No/Yes

#### 2.1.2 Nepal Diabetes Dataset

A significant contribution of this work is the introduction of a private dataset collected in Nepal. Data was obtained through voluntary participation after informed consent. The dataset comprises 1057 records. Unlike the UCI dataset, which emphasises both demographics and symptoms, it contains 60.5% non diabatic and 39.5% diabetic samples. The features of the Nepal Diabetes dataset are summarised in Table 2.

**TABLE 2:** Summarises the features of the Nepal Diabetes dataset.

Attribute	Data type	Possible values
Age	Numeric	20–65
Sex	Categorical	Female/ ale
Polyuria	Categorical	No/Yes
Polydipsia	Categorical	No/Yes
Sudden weight loss	Categorical	No/Yes
Weakness	Categorical	No/Yes
Polyphagia	Categorical	No/Yes
Genital thrush	Categorical	No/Yes
Visual blurring	Categorical	No/Yes
Itching	Categorical	No/Yes
Irritability	Categorical	No/Yes
Delayed healing	Categorical	No/Yes
Partial paresis	Categorical	No/Yes
Muscle stiffness	Categorical	No/Yes
Alopecia	Categorical	No/Yes
Obesity	Categorical	No/Yes
Class	Categorical	Negative/Positive

### 2.2 Data Preprocessing

In the Diabetes UCI dataset, categorical attributes such as yes/no, male/female and positive/negative were directly converted into binary values (1 and 0), with the prediction target defined as the ‘class’ column. In contrast, the Nepalese dataset required a more extensive cleaning pipeline because of bilingual entries in both English and Nepali (Devanagari script). All Nepali responses were first translated into their English equivalents, after which categorical responses and numeric values were uniformly encoded into binary or numeric form. Columns were coerced into numeric types, invalid entries were removed, and the target variable was identified as ‘diabetic’.

To ensure reproducibility, fixed random seeds were applied across all Python libraries and machine learning frameworks, and deterministic execution settings were enforced where possible. Data for both datasets was imported from CSV format, with features separated from their corresponding labels.

To prevent data leakage, all partitioning into training, validation and testing subsets was performed before any preprocessing operations that could learn from the data. For the UCI dataset, a stratified 70/30 split was first used to preserve class proportions; the 30% subset was then further divided into 10% validation and 20% hold‐out test sets. Standardisation parameters required for the DNN model—such as the mean and standard deviation used in z‐score normalisation—were computed only from the training subset. The hold‐out test set remained untouched throughout model development and was accessed only during the final evaluation.

In the Nepalese dataset, evaluation was conducted using stratified 5‐fold cross‐validation. For each fold, preprocessing steps such as encoding and normalisation were fit exclusively on the training portion of that fold and then applied to the corresponding validation subset, ensuring a leakage‐free assessment. Standardisation was required only for the DNN inputs; XGBoost was trained on the original encoded features.

### 2.3 Models

A hybrid ensemble of extreme gradient boosting (XGBoost) and a deep neural network (DNN) was employed to implement the proposed diabetes prediction system. All hyperparameters and architectures were optimised on the UCI dataset and then applied unchanged to the Nepal diabetes dataset within each fold of the 5‐fold cross‐validation.

#### 2.3.1 XGBoost

An XGBoost classifier was trained using one hundred boosting rounds and a learning rate of 0.1. Model performance was evaluated using logarithmic loss, and a fixed random seed was applied to ensure reproducibility. The data were provided to the model using XGBoost's optimised matrix structure. To mitigate overfitting, early stopping was employed, halting training if the validation log loss did not improve for twenty consecutive rounds. Final predictions were generated using the model state from the best‐performing iteration to ensure optimal predictive performance.

#### 2.3.2 Deep Neural Networks

Input features were standardised prior to training. The DNN architecture consisted of a fully connected layer with 64 rectified linear units, followed by a dropout layer with a rate of fifty percent. A second fully connected layer with 32 rectified linear units was then applied, leading to a single output neurone with a sigmoid activation function. Model training was conducted for a maximum of one hundred epochs using mini‐batches of 16 samples. To prevent overfitting, early stopping was employed based on validation loss, with training halted if no improvement was observed for ten consecutive epochs, and the model parameters from the best‐performing epoch were automatically restored.

#### 2.3.3 Ensemble

We used a soft‐voting ensemble to make our final predictions. This method averaged the probabilities from both an XGB and a DNN model, with a 0.5 threshold used to convert the averaged probabilities into binary outcomes.

This ensemble capitalised on XGB's ability to handle complex feature interactions and the DNNs' capacity for abstract representation learning, resulting in more reliable and consistent predictions than either model could achieve on its own.

## 3 Results and Discussion

This section presents the results and discussion of the proposed XGB–DNN hybrid ensemble for early‐stage diabetes prediction. First, we evaluated the performance of the individual models and the hybrid ensemble on both datasets. Then, we analysed their confusion matrices and training dynamics to illustrate classification behaviour and learning stability.

The models were evaluated using several key metrics: AUC, ACC, precision (PRE), recall and F1‐score.

For the UCI dataset, the models were trained and evaluated using a 70:10:20 stratified split for training, validation and testing, respectively, to preserve class distribution.

For the Nepal diabetes dataset, the same model architectures were applied, but evaluation was performed using stratified 5‐fold cross‐validation. For each fold, ACC and AUC were calculated, and the overall performance was summarised using the mean and standard deviation (SD) across all folds. This approach ensures a robust assessment of the model's generalisation ability on a dataset with potentially similar features and a larger size compared to the UCI dataset.

### 3.1 Diabetes UCI Dataset Results

Table 3 presents the performance metrics of XGB, DNN and the hybrid ensemble on the Diabetes UCI dataset, the hybrid ensemble outperformed the individual models in terms of ACC, recall, F1‐score and AUC.

**TABLE 3:** Performance metrics on the UCI dataset.

Model	ACC	PRE	Recall	F1	AUC
XGB	98%	0.98	0.98	0.98	0.99
DNN	97%	0.97	0.98	0.98	0.99
**Hybrid ensemble**	**99%**	**0.98**	**1.00**	**0.99**	**1.00**

**FIGURE 2:**
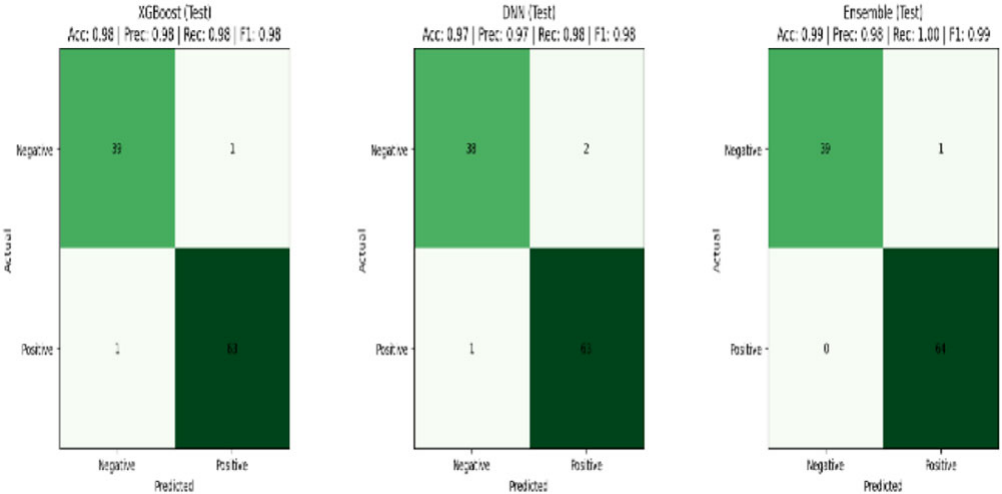
Shows the confusion matrices of XGB, DNN and the hybrid ensemble on the UCI dataset.

**FIGURE 3:**
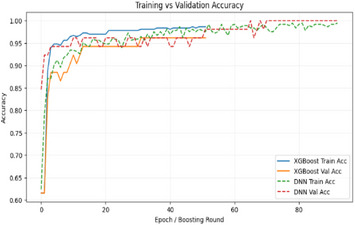
Training dynamics of the models on the UCI dataset. DNN training and validation loss across epochs (left) and XGB (right).

The corresponding confusion matrices are shown in Figure 2. The training dynamics for both DNN and XGB are illustrated in Figure 3. The DNN training and validation loss curves across epochs demonstrate smooth learning progression and generalisation, while the XGB training and test logloss curves across boosting rounds show a rapid drop in training loss and a more gradual decrease in test loss, reflecting stepwise convergence and generalisation to unseen data.

**FIGURE 4:**
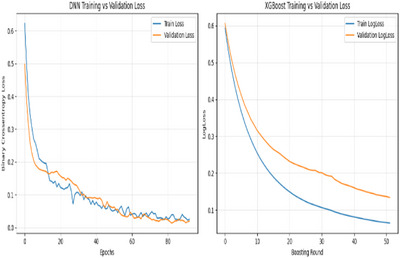
Training and validation accuracy curves for the DNN and XGB models on the UCI dataset.

**FIGURE 5:**
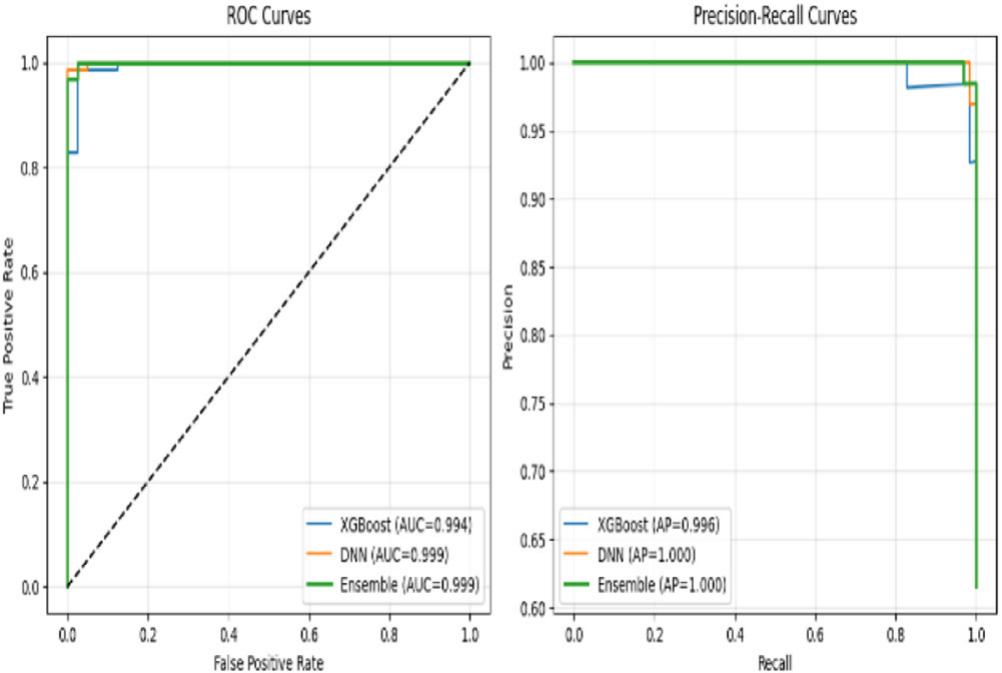
Comparative evaluation of UCI dataset models: ROC curves with AUC (left) and precision recall curves (right) for XGB, DNN and ensemble.

Similarly, Figure 4 presents the training and validation accuracy curves, highlighting the DNN's learning progression across epochs as well as XGB's convergence behaviour across boosting rounds. Additionally, comparative evaluation using ROC and PR curves is shown in Figure 5.

The near‐perfect AUC on the UCI dataset is expected due to the binary classification task with highly separable features, consistent with prior studies reporting similar results [11].

### 3.2 Nepal Diabetes Dataset Results

The Nepal dataset was evaluated with stratified 5‐fold cross‐validation. For each fold, accuracy (ACC) and area under the curve (AUC) were calculated for the individual models and the hybrid ensemble. The fold‐wise outcomes are displayed in Table 4.

**TABLE 4:** Reports the fold‐wise metrics for XGB, DNN and the hybrid ensemble on the Nepal Diabetes Dataset.

Fold	XGB Acc	XGB AUC	DNN Acc	DNN AUC	Ensemble Acc	Ensemble AUC
1	0.9057	0.9708	0.9528	0.9853	0.9481	0.9793
2	0.9147	0.9665	0.9384	0.9756	0.9336	0.9755
3	0.9005	0.9660	0.8768	0.9446	0.8957	0.9660
4	0.8863	0.9368	0.8863	0.9453	0.9005	0.9479
5	0.8673	0.9344	0.8673	0.9183	0.8815	0.9319
Mean ± SD	0.8949 ± 0.0166	0.9549 ± 0.0159	0.9043 ± 0.0346	0.9538 ± 0.0240	0.9119 ± 0.0249	0.9601 ± 0.0201

Fold‐wise variation in performance is expected due to differences in the specific training and test splits. Importantly, the hybrid ensemble's primary advantage lies in reducing variance across folds, rather than guaranteeing per‐fold superiority. This demonstrates the ensemble's ability to provide more stable and reliable predictions on datasets with potentially heterogeneous characteristics.

### 3.3 Discussion

Our analysis of the hybrid XGB–DNN ensemble model for early‐stage diabetes prediction demonstrates its superior performance, robustness and generalisation across two distinct datasets. The ensemble consistently outperformed its individual components—XGB and DNN—in terms of accuracy and AUC, confirming its ability to leverage complementary strengths for more stable and reliable predictions.

On the UCI dataset, evaluated using a 70:10:20 stratified hold‐out split, the ensemble achieved 99.04% accuracy, an AUC of 1.00 and perfect recall (1.00). Preserving class distribution across training, validation and testing sets reduced bias, while rigorous preprocessing—including handling missing values, encoding categorical features and normalising numerical features—contributed to the model's strong performance. Notably, while the CNN model reported in 2021 achieved 100% precision, our ensemble's 100% recall is more clinically important for early‐stage diabetes screening, as it ensures all potential cases are identified and false negatives are minimised.

For the Nepal diabetes dataset, evaluated using stratified 5‐fold cross‐validation, the ensemble achieved an average accuracy of 91.2% (±2.5%) and an AUC of approximately 0.96 across all folds. Despite challenges such as bilingual entries and heterogeneous patient subgroups, the ensemble demonstrated remarkable stability, with low fold‐to‐fold variation (2%–3% SD), effectively mitigating performance drops in more heterogeneous folds.

The ensemble's consistent performance across both datasets highlights its strong generalisation capability. Measures such as early stopping and dropout for the DNN, alongside early stopping for XGB, prevented overfitting and enhanced robustness and reliability across diverse, real‐world populations. Collectively, these results underscore the ensemble's suitability for early‐stage diabetes screening, where high recall and stable performance are critical for timely clinical intervention.

## 4 Conclusion

This study successfully developed an XGBoost–DNN hybrid ensemble for early‐stage diabetes risk prediction, achieving remarkable performance across both datasets. The model attained 99% accuracy on the Diabetes UCI dataset and 91% accuracy on the Nepal diabetes dataset, demonstrating higher recall, more stable performance and stronger generalisation compared to standalone classifiers and existing benchmark models. These results highlight the effectiveness of combining machine learning and deep learning techniques within a single hybrid framework.

The study highlights several important insights. First, the hybrid ensemble effectively leverages the complementary strengths of XGBoost and DNNs, capturing both structured patterns and complex nonlinear relationships in the data. Second, the model generalises well to real‐world, self‐reported non‐clinical data, demonstrating practical applicability beyond controlled benchmark datasets. Third, by reducing false negatives, the model ensures clinical relevance, supporting timely identification of early‐stage diabetes and enabling improved patient outcomes.

Overall, the findings confirm the potential of hybrid ML‐DL frameworks as low‐cost, scalable and reliable tools for early diabetes detection, particularly in resource‐constrained settings. Future work will focus on evaluating the model on larger, multi‐centre datasets to further validate generalisability, developing a lightweight web‐based application to enhance accessibility for clinicians and patients, and exploring adaptive ensemble strategies to further improve robustness and predictive accuracy.

## Author Contributions

**Rachana Katuwal KC**: conceptualization, methodology, software, validation, formal analysis, writing – original draft. **Su Yang**: validation, writing – review & editing, supervision.

## Funding

The authors have nothing to report.

## Conflicts of Interest

The authors declare no conflicts of interest.

## Data Availability

The UCI diabetes dataset used in this study is publicly available via kaggle. The Nepal diabetes dataset is available from the author upon reasonable request.
